# Supplementary material for: 4polar3D single molecule imaging of 3D orientation in dense actin networks using ratiometric polarization splitting
Source: Nat Commun. 2026 Mar 19;17:4246. doi: 10.1038/s41467-026-70852-y (PMC13168503; doi:10.1038/s41467-026-70852-y)
Supplement: Supplementary file 2 — Reporting Summary [file 41467_2026_70852_MOESM2_ESM.pdf]

nature portfolio

Corresponding author(s): Sophie Brasselet

Last updated by author(s): Feb 16, 2026

Reporting Summary

Nature Portfolio wishes to improve the reproducibility of the work that we publish. This form provides structure for consistency and transparency in reporting. For further information on Nature Portfolio policies, see our Editorial Policies and the Editorial Policy Checklist.

Statistics

For all statistical analyses, confirm that the following items are present in the figure legend, table legend, main text, or Methods section.

n/a	Confirmed	
☐	☒	The exact sample size (*n*) for each experimental group/condition, given as a discrete number and unit of measurement
☐	☒	A statement on whether measurements were taken from distinct samples or whether the same sample was measured repeatedly
☐	☒	The statistical test(s) used AND whether they are one- or two-sided<br>*Only common tests should be described solely by name; describe more complex techniques in the Methods section.*
☒	☐	A description of all covariates tested
☒	☐	A description of any assumptions or corrections, such as tests of normality and adjustment for multiple comparisons
☐	☒	A full description of the statistical parameters including central tendency (e.g. means) or other basic estimates (e.g. regression coefficient) AND variation (e.g. standard deviation) or associated estimates of uncertainty (e.g. confidence intervals)
☐	☒	For null hypothesis testing, the test statistic (e.g. *F*, *t*, *r*) with confidence intervals, effect sizes, degrees of freedom and *P* value noted<br>*Give P values as exact values whenever suitable.*
☒	☐	For Bayesian analysis, information on the choice of priors and Markov chain Monte Carlo settings
☒	☐	For hierarchical and complex designs, identification of the appropriate level for tests and full reporting of outcomes
☒	☐	Estimates of effect sizes (e.g. Cohen's *d*, Pearson's *r*), indicating how they were calculated

Our web collection on statistics for biologists contains articles on many of the points above.

Software and code

Policy information about availability of computer code

Data collection	Data collection was performed using the software provided by the Hamamatsu company (HCImage - Image Acquisition software).
Data analysis	The MATLAB code used to analyse the data is available on GitHub at https://github.com/CessVala/4polar3D_SMOLM, which includes a manual and installation instructions. The Python code used for the Monte Carlo simulation is available on GitHub at https://github.com/CessVala/4polar3D_SMOLM_simulation.

For manuscripts utilizing custom algorithms or software that are central to the research but not yet described in published literature, software must be made available to editors and reviewers. We strongly encourage code deposition in a community repository (e.g. GitHub). See the Nature Portfolio guidelines for submitting code & software for further information.

Data

Policy information about availability of data

All manuscripts must include a data availability statement. This statement should provide the following information, where applicable:

- Accession codes, unique identifiers, or web links for publicly available datasets
- A description of any restrictions on data availability
- For clinical datasets or third party data, please ensure that the statement adheres to our policy

The 4polar3D raw image stacks generated in this study are available upon request from the corresponding author (due to the large size of the files, 150-250 GB). A subset of data (2 GB), as well as the processed orientation/detection parameters from single-molecule data generated in this study are available in the Source Data

file (https://doi.org/10.6084/m9.figshare.28890470). Processed data for example ROIs generated in this study are available for download at https://github.com/CessVala/4polar3D_SMOLM (TestData folder), with explanations provided in the README.md file.

Research involving human participants, their data, or biological material

Policy information about studies with human participants or human data. See also policy information about sex, gender (identity/presentation), and sexual orientation and race, ethnicity and racism.

Reporting on sex and gender	unknown Information
Reporting on race, ethnicity, or other socially relevant groupings	unknown Information
Population characteristics	unknown Information
Recruitment	unknown Information
Ethics oversight	unknown Information

Note that full information on the approval of the study protocol must also be provided in the manuscript.

Field-specific reporting

Please select the one below that is the best fit for your research. If you are not sure, read the appropriate sections before making your selection.

☒ Life sciences ☐ Behavioural & social sciences ☐ Ecological, evolutionary & environmental sciences

For a reference copy of the document with all sections, see nature.com/documents/nr-reporting-summary-flat.pdf

Life sciences study design

All studies must disclose on these points even when the disclosure is negative.

Sample size	Typically 3-5 cells are measured for each study (lamellipodia, podosomes), and about 3 studies were performed at different days. The reproducibility of the measurements allowed to conclude that such sample is sufficient. More than 10 regions per cells were typically extracted for the lamellipodia study, while hundreds of podosomes were extracted per cell for the podosome study, providing enough statistics for comparisons.
Data exclusions	The exclusion of some of the 4polar3D single molecule detections is explained in the Methods section: detections corresponding to eta and delta reaching extreme values are discarded. The reason, explained in the manuscript, is that a larger data bias is introduced for these extreme cases (this bias is quantified in the Monte Carlo simulations given in Fig. 2)
Replication	Measurements were seen to be reproducible (comparison between groups of 3-5 cells measured at 3 different days show very similar histograms and image behaviors).
Randomization	There are no specific experimental groups studied in this work, that justifies the use of random allocation.
Blinding	Blinding is not relevant in this study since no specific comparison is made between samples or with a given control sample.

Reporting for specific materials, systems and methods

We require information from authors about some types of materials, experimental systems and methods used in many studies. Here, indicate whether each material, system or method listed is relevant to your study. If you are not sure if a list item applies to your research, read the appropriate section before selecting a response.

Materials & experimental systems

n/a	Involved in the study	
☒	☐	Antibodies
☐	☒	Eukaryotic cell lines
☒	☐	Palaeontology and archaeology
☒	☐	Animals and other organisms
☒	☐	Clinical data
☒	☐	Dual use research of concern
☒	☐	Plants

Methods

n/a	Involved in the study	
☒	☐	ChIP-seq
☒	☐	Flow cytometry
☒	☐	MRI-based neuroimaging

Eukaryotic cell lines

Policy information about cell lines and Sex and Gender in Research

Cell line source(s)	Monocytes from healthy subjects (HS) were provided by Etablissement Français du Sang, Toulouse, France, under contract 21RB2025-028-R and 21RB2025-031-R. According to articles L12434 and R124361 of the French Public Health Code, the contract was approved by the French Ministry of Science and Technology (agreement number AC 2009921). Written informed consents were obtained from the donors before sample collection.<br>B16-F1 cells were a gift from Klemens Rottner, Technische Universität Braunschweig, Germany, and were originally from ATCC (CRL- 6323).
Authentication	The B16-F1 cell line was not authenticated.
Mycoplasma contamination	The cell line was not tested for mycoplasma contamination.
Commonly misidentified lines (See ICLAC register)	N/A

Plants

Seed stocks	N/A
Novel plant genotypes	N/A
Authentication	N/A
